# Shape Memory Polymer Foam with Programmable Apertures

**DOI:** 10.3390/polym12091914

**Published:** 2020-08-25

**Authors:** Mario Walter, Fabian Friess, Martin Krus, Seyed Mohammad Hassan Zolanvari, Gunnar Grün, Hartmut Kröber, Thorsten Pretsch

**Affiliations:** 1 Fraunhofer Institute for Applied Polymer Research IAP, Geiselbergstr. 69, 14476 Potsdam, Germany; mario.walter@iap.fraunhofer.de (M.W.); fabian.friess@iap.fraunhofer.de (F.F.); 2 Fraunhofer Institute for Building Physics IBP, Fraunhoferstraße 10, 83626 Valley, Germany; martin.krus@ibp.fraunhofer.de (M.K.); seyed.zolanvari@ibp.fraunhofer.de (S.M.H.Z.); gunnar.gruen@ibp.fraunhofer.de (G.G.); 3 Fraunhofer Institute for Chemical Technology ICT, Joseph-von-Fraunhofer-Straße 7, 76327 Pfinztal, Germany; hartmut.kroeber@ict.fraunhofer.de

**Keywords:** foam, programmable material, polyester urethane urea, actuation, construction, air slot

## Abstract

In this work, a novel type of polyester urethane urea (PEUU) foam is introduced. The foam was produced by reactive foaming using a mixture of poly(1,10–decamethylene adipate) diol and poly(1,4–butylene adipate) diol, 4,4′-diphenylmethane diisocyanate, 1,4–butanediol, diethanolamine and water as blowing agent. As determined by differential scanning calorimetry, the melting of the ester-based phases occurred at temperatures in between 25 °C and 61 °C, while the crystallization transition spread from 48 °C to 20 °C. The mechanical properties of the foam were simulated with the hyperplastic models Neo-Hookean and Ogden, whereby the latter showed a better agreement with the experimental data as evidenced by a Pearson correlation coefficient R² above 0.99. Once thermomechanically treated, the foam exhibited a maximum actuation of 13.7% in heating-cooling cycles under a constant external load. In turn, thermal cycling under load-free conditions resulted in an actuation of more than 10%. Good thermal insulation properties were demonstrated by thermal conductivities of 0.039 W·(m·K)^−1^ in the pristine state and 0.052 W·(m·K)^−1^ in a state after compression by 50%, respectively. Finally, three demonstrators were developed, which closed an aperture or opened it again simply by changing the temperature. The self-sufficient material behavior is particularly promising in the construction industry, where programmable air slots offer the prospect of a dynamic insulation system for an adaptive building envelope.

## 1. Introduction

Shape memory polymers (SMPs) are stimuli-responsive materials. [1,2,3,4,5] Once thermomechanically treated (programmed), they maintain a temporary shape until the shape memory effect (SME) is triggered. The activation of the so-called one-way shape memory effect (1W SME) results in the almost complete recovery of the original shape. The molecular network architecture supports the advantageous thermomechanical behavior of SMPs. Molecular chains and cross-linking points, whose nature can be physical or chemical, define the structure. In segmented polyurethane (PU), the exact chemical composition has a significant influence on phase morphology while the ratio of hard to soft segments affects phase separation [6,7,8]. Today, physically cross-linked, phase segregated block copolymers like poly(ester urethanes) (PEUs) belong to the most promising SMP families [9,10,11,12,13,14,15,16,17,18]. Here, switching can be accomplished when passing the glass transition (*T*_trans_ = *T_g_*) or the melting transition (*T*_trans_ = *T_m_*) of the polyester soft segment [19,20]. Advantageously, *T*_trans_ can be synthetically adjusted in a wide temperature range, e.g., by changing the molecular weight of the soft segment and thereby the cross-link density and the chemical composition [21,22,23,24]. The hard segment counterparts act as physical cross-links in the rubbery soft segment matrix and are characterized by a higher thermal transition temperature.

It has been known for some years now, that SMPs can also be thermomechanically treated in such a way, that they can move back and forth between two states depending on the ambient temperature [25,26,27]. Importantly, this two-way shape memory effect (2W SME) can be triggered repeatedly under stress-free conditions without the need for a re-programming in between. The development of the new programming routes constituted a significant step forward compared with the conventional 1W shape memory technology, since materials were obtained with a “built-in” IF...THEN...ELSE switching function that was available in many consecutive cycles. Thus, the proof-of-principle for a new generation of programmable materials could be achieved, changing shape upon temperature variation. Mechanistically, directional crystallization and melting of the switching segment enables the actuation of programmed SMPs, same as entropy elasticity, as well-known from actuation experiments under a constant external load [24,28,29]. Against this background, recent studies focused on the development of concepts to introduce bidirectional actuation to novel polymeric systems [30,31] and addressed several technological fields like biomedical applications [32], soft robotics [33] and 4D printing [34].

While the 1W SME of polyurethane-based foams has been thoroughly studied for a while now [35,36,37,38], there are only few publications, in which the potential of foams with two-way actuation capability is presented. For instance, Mather and co-workers employed a modified porogen-leaching technique to fabricate highly porous scaffolds from poly(ε–caprolactone) (PCL) and poly(ethylene glycol) (PEG), which exhibited bidirectional actuation under a constant external load with a maximum actuation strain of 15% at temperatures between −20 °C and 80 °C [39]. In another approach, Li’s group prepared a syntactic foam with two-way shape memory properties from cross-linked PCL filled with glass microspheres, which showed under external load a temperature-dependent actuation by 10% [40]. In a third work, Zharinova et al. proved that water-blown polyurethane foams with PCL-based switching segments actuate at temperatures between 10 °C and 49 °C under load-free conditions [41]. It is noteworthy that the above approaches successfully transferred the thermoreversible effects to cellular systems, but these works had no relation to applications such as construction.

In the European Union, the contribution of buildings to the final energy consumption is estimated to be 40% and responsible for 36% of the total CO_2_ emission [42]. The temperature conditioning of building interiors is a significant source of this consumption. Traditional insulation materials like glass or mineral wool, extruded polystyrene (XPS), expanded polystyrene (EPS) or polyurethane rigid foam with specific insulation properties are used to minimize heat flow through the walls. But especially during long hot days or summer periods the building envelope as well as the interior can reach temperatures which make cooling necessary. As a matter of fact, switchable insulating systems have been developed with the aim to enhance indoor thermal environment and simultaneously reduce the energy demand in buildings. A greater number of materials and constructions have been investigated that can switch between an insulating and a thermally conducting state [43,44]. These approaches include e.g., multilayered walls [45], adaptive glazing systems [46] or moving shell structures [43]. The energy saving potential of such technologies and concepts has been demonstrated using different simulation models and was experimentally validated [44,47,48]. Furthermore, current studies can be found in which shape memory polymers are utilized for adaptive shading systems [49,50].

Following a different approach, we report here on the development of a novel polyester urethane urea (PEUU) foam with two-way shape memory properties and its application potential in the building industry. For this purpose a reactive foaming process was developed and the chemistry of classical PEU synthesis employed. To be more precise, the PEUU foam was obtained when reacting a mixture of poly(1,10–decamethylene adipate) (PDA) and poly(1,4–butylene adipate) diol (PBA) with a diisocyanate and finally adding a second mixture of cross-linker and water as blowing agent. The choice of chemicals was made to ensure that the thermal specifications were met by the foam. These consisted of a limited temperature window for actuation with a maximum width of 30 °C at temperatures slightly above 20 °C. The mechanical behavior of the foam was studied and an attempt was made to identify a hyperelastic material model that would allow the material behavior of the foam to be simulated, e.g., under varying thermal or mechanical stresses or in combination with other materials, in so-called sandwich structures [51]. With the further emphasis to design an actuator, which can be integrated into insulation systems in buildings, the insulation properties were studied and foam demonstrators with programmable apertures were developed.

## 2. Materials and Methods

### 2.1. Materials

1,10–Decanediol, titanium(IV) isopropoxide (TTIP) and 4,4′–diphenylmethane diisocyanate (MDI) were purchased from Fisher Scientific (Schwerte, Germany) and diethanolamine (DEA) from Merck Millipore (Darmstadt, Germany). Adipic acid, 1,4–butanediol (BD) and the amine blowing catalyst 1,4–diazabicyclo[2.2.2]octane were received from Alfa Aesar (Kandel, Germany). The tin-free gelling catalyst K-Kat XC-B221 was kindly provided by Worlée (Hamburg, Germany). The surfactant Tegostab B8407 was obtained from Evonik (Essen, Germany). MDI was melted and decanted prior to use to remove solid residues and contaminants. All other materials were used as received. Deionized water was used as chemical blowing agent.

The synthesis process for the production of the foam was as follows. Poly(1,10–decamethylene adipate) diol (PDA) and poly(1,4–butylene adipate) diol (PBA) were prepared in titanium(IV)-catalyzed polycondensation reactions with adipic acid [52,53]. The PEUU foam was synthesized in a one-pot two step reaction from the abovementioned diols in the presence of a slight excess of MDI with a ratio of NCO/OH = 1.03. Therefore, a polyester mixture of PDA and PBA was produced in a ratio of 6.5:1, placed into a 550 mL polypropylene beaker and dried overnight at 85 °C in a vacuum oven. After 12 h, the polymer melt was removed and placed on a heating plate, where it was kept for 2 min at 80 °C, while being stirred with an overhead stirrer. After addition of surfactant (1.0 pphp) and gelling catalyst (0.3 pphp), molten MDI (36.7 pphp) was added under vigorous stirring. BD (1.0 pphp), DEA (2.0 pphp) and a mixture of deionized water (1.3 pphp) and blowing catalyst (0.1 pphp) were added at high stirring speed to finalize the reaction and initiate the foaming process. As soon as significant foaming occurred, the stirrer was removed to enable the free rise of the foam. The freshly prepared foam was kept overnight at 23 °C for post-curing.

### 2.2. Sample Preparation

Different sized samples were taken from the center of the foam using a band saw (HBS230HQ, Holzmann Maschinen GmbH, Haslach, Austria). To characterize their properties rectangular pieces with square cross sections of approximately 10 mm × 10 mm were prepared and cube-shaped samples were then cut out with a scalpel. A digital caliper was used to verify sample dimensions before measuring. Cuboidal samples were obtained by cutting slices of the foam perpendicular or parallel to rise direction followed by removing the edge regions. Samples with a height of 2 cm and a base of 4 cm × 4 cm were used to evaluate the thermal properties. Foam cuboids with dimensions of 20 mm × 20 mm × 80 mm, 20 mm × 20 mm × 40 mm and 10 mm × 50 mm × 45 mm were employed for demonstrator construction.

### 2.3. Characterization Methods

The molecular weights of polyester diols were calculated from the acid value and hydroxyl value of the corresponding polyols determined from titration according to standardized procedures using a TitroLine 7000 (SI Analytics, Mainz, Germany) [54,55].

The density of the foam was determined with an analytical balance (AEJ 100, Kern & Sohn GmbH, Balingen-Frommern, Germany). The cube-shaped samples had a dimensioning of 9.5 cm × 10.5 cm × 9.5 cm. In total five measurements were carried out on different samples and the results were averaged. The sample density was calculated as the ratio between the weight and volume of every sample. 

Micro-computed tomography (µCT) using a Skyscan 2211 (Bruker, Kontrich, Belgium) with X-ray source XWT-160-TC (X-Ray WorX GmbH, Garbsen, Germany) and a detector CCD camera MX 11002 (XIMEA GmbH, Münster, Germany) was employed to evaluate the cellular structures, pore size distribution and the interconnectivity of the pores. Accelerating voltage and current for the X-ray source tube were set as 90 kV and 100 mA, respectively. The reconstruction of foam elements and calculations were done with NREcon 1.7.4.2 (Bruker, Kontrich, Belgium) and InstaRecon CBR 2.0.3.5 (InstaRecon, Champaign, IL, USA). The analysis of the data was accomplished with CT Analyser 1.20.3.0 (Bruker, Kontrich, Belgium). Images of the foam structure were created using the software CTVox 3.3.0 (Bruker, Kontrich, Belgium). The cell size distribution and the interconnectivity of the cells was estimated by immersing a foam sample into 1–octanol for 24 h to fill interconnected cells while closed cells remained inaccessible. The subsequent µCT measurement gave images only showing the closed cells.

The closed cell content *X_cc_* was estimated using Equation (1), where φ*_cc_* is the volume fraction of closed cells and φ*_c_* is the volume fraction of all cells detected.

(1) $$X_{cc}=\frac{\varphi_{cc}}{\varphi_{c}}\;\times 100\%\;.$$

Additionally, the closed cell ratio was estimated using a self-designed rapid test method. Herein, buoyancy of cuboidal samples in a silicon oil was measured after a soaking time of 72 h. Special grade low viscosity silicon oil (Ebesil Öl B 0,65, Quax Gmbh, Otzberg, Germany) was used to ensure the complete flooding of the open cells.

The gel fraction was determined by an extraction procedure utilizing tetrahydrofuran (THF) as solvent according to procedures described elsewhere [56]. The gel fraction *GF* (%), was calculated as

(2) $$GF=\;\frac{m_{2}}{m_{1}}\;\times 100\%$$

where *m*_1_ and *m*_2_ are the masses of dried PU foam before and after the extraction, respectively. The reported value for *GF* corresponded to the mean value of five measurements.

The thermal conductivity was determined according to DIN EN 12664 using a guarded hot plate apparatus [57]. The measurements were carried out with a temperature of about 30 °C on the upper side and a temperature of about 10 °C on the lower side of the sample. The dimensions of the sample were reduced compared to the standard, due to the limited sample volume available. A sample with a cross-sectional area of 150 mm × 150 mm and a height of 20 mm was obtained by combining several smaller sized samples. In this way, the thermal conductivity could be determined at least in good approximation. The thermal resistance *R* was calculated as the quotient of sample height *h* and thermal conductivity *λ*, according to:

(3) $$R=\;\frac{h}{\lambda }\;.$$

The thermal properties of polyester diols and PEUU foam were investigated by differential scanning calorimetry (DSC) using a Q100 DSC (TA Instruments, New Castle, DE, USA). Samples were weighing approximately 5 mg in case of polyols and approximately 3 mg in case of foam. In every DSC measurement, two thermal cycles were run using heating and cooling rates of 10 K·min^−1^. The temperature area ranged from −10 °C to 110 °C for the polyester diols and from −80 °C to 80 °C in case of the PEUU foam. A first cycle was run to eliminate the thermal history, while measurement data of the second cycle was used to investigate the phase transitions. In every measurement cycle, the sample was kept for 2 min at the highest and lowest temperature, respectively. For the foam an additional measurement cycle was conducted applying a reduced heating and cooling rate of 1 K·min^−1^ to evaluate the phase transitions under conditions closer to an application scenario.

Dynamic mechanical analysis (DMA) was used to investigate the thermomechanical properties, actuation behavior and thermal expansion of the foam. The experiments were carried out with a Q800 DMA from TA Instruments (New Castle, DE, USA). In any case, cube-shaped samples with an edge length of about 10 mm were used. The temperature dependent storage modulus of the PEUU foam was determined at a frequency of 2 Hz, applying a static force of 0.25 N and an oscillating strain of 3%. The foam sample was cooled down to −80 °C with a rate of 3 K∙min^−1^, followed by an isothermal equilibration step of 20 min. The measurement was then started and the foam was heated to 80 °C with a rate of 3 K∙min^−1^. Thermomechanical testing was carried out to determine the mechanical behavior, hardness and compressive strength of the foam at different temperatures. The experiments were run in controlled force mode and were performed at 25 °C, 35 °C, 45 °C, 55 °C, 65 °C and 75 °C, using a loading rate of 5 N·min^−1^ until the maximum force was achieved. The maximum force was 15 N for 25 °C, 35 °C and 45 °C and was adjusted to 13 N for the measurement at 55 °C and to 12 N for the experiments at 65 °C and 75 °C in order to avoid large compressive strain values above 70%. After 15 min at constant load, unloading was accomplished with a rate of 5 N·min^−1^ whereupon only a contact load of 0.1 N remained on the foam. The measurements were performed one after another, separated by an equilibration time of 15 min, before heating to the next temperature with a rate of 5 K∙min^−1^ was carried out.

**Figure 1:**
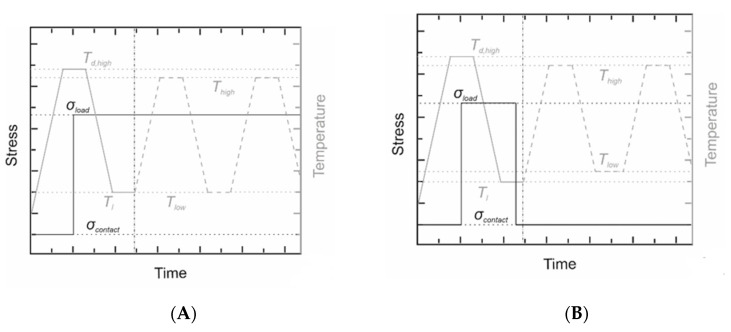
Thermomechanical investigation of PEUU foam to characterize bidirectional actuation under constant load (**A**) and load-free conditions (**B**). A vertical dotted line was inserted to illustrate the transition from programming to actuation.

The actuation of the foam in relation to its height was investigated in cyclic thermomechanical measurements both under constant load and load-free conditions. The associated measurement specifications are provided by Figure 1.

In case of constant load conditions (Figure 1A), the sample was heated to the deformation temperature *T_d_* = 70 °C, kept there for 30 min and loaded with 2 N followed by temperature conditioning for 10 min. Adjacently, the sample was cooled to *T_l_* = 20 °C and equilibrated for 25 min before the temperature was cycled between *T*_high_ = 65 °C and *T*_low_ = 20 °C, followed by an equilibration time of another 25 min. Heating and cooling rates were set to 5 K∙min^−1^ and a loading rate of 5 N∙min^−1^ was applied. To verify the influence on bidirectional actuation, additional experiments with varying temperature ranges, temperature conditioning times as well as different static forces were performed. In all cases the thermal cycling between *T*_low_ and *T*_high_ was repeated once or twice except for long-term measurements. Here, eleven cycles between *T*_low_ = 20 °C and *T*_high_ = 65 °C were measured followed by another six cycles between *T*_low_ = 25 °C and *T*_high_ = 55 °C to verify the stability of actuation. 

The reversible strain *ε*_rev_ is the key parameter when studying thermoreversible actuation in polymers. In case of foams it can be defined as the absolute change in sample height according to Equation (4).

(4) $$\varepsilon_{rev}\left(N\right)=\;-\frac{h_{\mathrm{low}}\left(N\right)-\;h_{\mathrm{high}}\left(N\right)}{h_{\mathrm{low}}\left(N\right)}\;\times \;100\%\;.$$

Herein, *h*_high_(*N*) and *h*_low_(*N*) are the sample’s height at *T*_high_ *h* and *T*_low_ in the course of actuation. In addition, measurements under load-free conditions were performed (Figure 1B). In this case, samples were heated to the deformation temperature *T_d_* = 70 °C, equilibrated for 20 min under isothermal conditions and loaded with 2 N, 4 N or 6 N followed by another 20 min of isothermal equilibration. After cooling to *T*_low_ = 15 °C, isothermal equilibration for 20 min and unloading, samples were subjected to repeated thermal cycling with varying *T*_high_ and *T*_low_, while continuously applying a contact force of 0.05 N. Rates for heating/cooling and loading/unloading were the same as under constant load conditions. 

The thermal expansion behavior of the foam was studied in a separate DMA measurement. Therefore, a sample of pristine PEUU foam was cycled three times each in a temperature interval of 45 °C applying three different upper and lower temperatures *T*_1_ and *T*_2_, namely −20 °C, 0 °C and 20 °C. The sample was first heated to the upper temperature *T*_2_ and then equilibrated for 20 min. After cooling to the lower temperature *T*_1_ and equilibration for 20 min, the thermal cycling was repeated twice. Heating and cooling rates of 5 K·min^−1^ and a weak external load of 0.05 N were applied while the change in sample height was recorded. The thermal expansion coefficient α*_T_*_1/*T*2_ was calculated as

(5) $$\alpha_{T1/T2}=\;\frac{\Delta L\;}{L_{0}\cdot \Delta T}$$

where α*_T_*_1/*T*2_ is the mean thermal expansion of the foam in the interval between temperatures *T*_1_ and *T*_2_, Δ*L* is the change in height, *L*_0_ is the initial height and Δ*T* is the width of the temperature interval applied.

### 2.4. Demonstrators

Experimental studies on demonstrators with bigger sized samples having a volume of up to 32 cm³ were performed in a temperature chamber TH2700 (Thümler GmbH, Nürnberg, Germany) using self-designed sample holders. Initially, a sample was heated to *T_d_* = 70 °C, before a constant load was applied by placing a metal cylinder atop the foam. To achieve an adequate compression of at least 20%, the weight of the cylinder was adapted to the base area of the foam. The mass to area ratio was set to 200 g·cm^−2^, which corresponds to a stress of 19.6 kPa. Actuation of the foam was followed when cycling the temperature between *T*_high_ = 70 °C and *T*_low_ = 20 °C.

### 2.5. Numerical Simulation

The main objective of this part was to predict the mechanical behavior of the PEUU foam. The foam is presenting non-linear and rubber-like behavior, its stress-strain response is not linear, and the material can undergo large elastic deformations. Therefore, two hyperelastic models were used to predict its mechanical response:

- Neo-Hookean model
- Ogden model

The Neo-Hookean is the simplest hyperelastic model which is based on linear functions of the first strain invariants and has one material constant. For the current measurement with uniaxial compression, there are other hyperelastic models proposed in the literature. Accordingly, the following models were tested: Mooney-Rivlin [58,59] (a polynomial model), van der Waals [60] (a physical model), and Yeoh [61] (a reduced polynomial model). The results of these models were unstable at some temperatures mainly due to a higher number of constants and the only stable model at all temperatures was Neo-Hookean. The reason for choosing Ogden is the accuracy of the model. According to the literature, e.g., Reference [62], this model can predict the stress-strain response of shape memory polymers more accurately in comparison with other hyperelastic models. The Ogden strain energy function, which is used in this work, is of 1st order with two material constants. It should be noted that the 2nd and 3rd orders of the Ogden model were also tested. Unlike than presented in recent articles [63], a model of higher order led to unstable results, especially in larger strains. The reason for this instability was the higher number of model parameters, i.e., two parameters per order, which caused overfitting. Accordingly, the results of 1st order Ogden and Neo-Hookean are presented in this work.

Based on these models, the strain energy functions were computed and then analytical stresses were calculated in uniaxial compression mode. The material constants of the models were computed using a least-squares fit algorithm. These steps were performed with the commercial finite element software Abaqus (Dassault Systèmes, Vélizy-Villacoublay, France). The results were compared with the DMA measurements on the foam and the Pearson correlation coefficient R² was used to evaluate the model prediction. 

The strain energy function refers to the strain energy stored in a material per unit of the initial volume and the hyperelastic models predicted the strain energy function for the corresponding materials. Stress and strain were derived by differentiating the strain energy function. Rivlin showed this for incompressible and isotropic materials under uniaxial compression [59]:

(6) $$\frac{\sigma }{\lambda -\lambda^{-2}}=2\left[\frac{\partial W}{\partial I_{1}}-\frac{1}{\lambda }\frac{\partial w}{\partial I_{2}}\right]$$

where *σ*, *ε*, *W*, and *λ* are stress, principal deformation, strain energy function, and principal stretch ratio, respectively. In this equation, *W* is a function of three invariants *l_i_*:

(7) $$W=f\left(I_{1},I_{2},I_{3}\right)\;.$$

#### 2.5.1. Neo-Hookean Model

The Neo-Hookean model is micromechanical-based and its strain energy function was developed according to the physics of polymer chain networks. It represents a simplified case of the Mooney-Rivlin model [64]. In this case, *W* is derived based on the first strain invariant:

(8) $$W=C_{10\;}\left(\;I_{1}-3\right)+\;\frac{1}{D_{1}}{\left(\;J-1\right)}^{2}$$

where *C*_10_ is a temperature-dependent material constant, which is one half of the initial shear modulus, and *D*_1_ governs the compressibility with which the bulk modulus can be computed.

#### 2.5.2. Ogden Model

The Ogden strain energy function is derived based on principal stretches, which is in contrast with the invariant-based models [65]. This model probably is the most developed model [66] and its strain energy function is:

(9) $$W=\overset{n}{\underset{i=1}{\sum }}\frac{\mu_{i\;}}{\alpha_{i\;}}\;\left(\lambda_{1}^{\alpha_{i}}+\lambda_{2}^{\alpha_{i}}+\lambda_{3}^{\alpha_{i}}-3\right)\;+\;\overset{n}{\underset{j=1}{\sum }}\frac{1}{D_{j}}\;{\left(J-1\right)}^{2j}{}_{}.$$

Herein, *n* is a material parameter, *µ_n_* and *α_n_* are temperature-dependent material constants and *D_j_* governs the compressibility. Accordingly, the initial shear modulus can be derived by $\;\overset{n}{\underset{i=1}{\sum }}\mu_{i}$. This equation can be simplified in case of uniaxial compression, since *λ*_1_ = *λ* and *λ*_2_ = *λ*_3_ = *λ*^−1/2^. In this work, different values of *n* were tested and it was observed that only the 1st order was stable at all temperatures. Considering the 1st order, Ogden strain energy function is:

(10) $$W=\frac{\mu_{1}}{\alpha_{1}}\;\left(\lambda^{\alpha_{1}}+\;2\lambda^{-\alpha_{1}/2}-3\right)+\frac{1}{D_{1}}{\left(J-1\right)}^{2}{}_{}.$$

## 3. Results and Discussion

### 3.1. Polyester Diols

In a first step, two polyester diols which were later used as building blocks for the PEUU foam were synthesized employing a titanium-(IV)-catalyzed polycondensation reaction. The molecular weights of poly(1,10–decamethylene adipate) diol (PDA) and poly(1,4–butylene adipate) diol (PBA) were calculated with 3500 and 2200 g·mol^−^^1^. The obtained polyester diols were investigated by differential scanning calorimetry (DSC) and exhibited melting peak temperatures of 69 °C for PDA and signals at 47 °C and 52 °C for PBA [67], while the peak temperatures of crystallization were located at 57 °C for PDA and at 32 °C for PBA. For PDA a remarkably high affinity to crystallize was evidenced by a melting enthalpy above 140 J·g^−^^1^. Strong intermolecular interactions and the higher molar mass of PDA as associated with a higher probability for the occurrence of entanglements obviously supported a high melt viscosity at a reaction temperature of 80 °C. Addition of PBA as a co-polyol lowered the viscosity, thus serving for an improved mixing with low viscosity components as isocyanate, cross-linkers and blowing agent. The analytical results are summarized in Table 1.

### 3.2. Foam Structure

**Figure 2:**
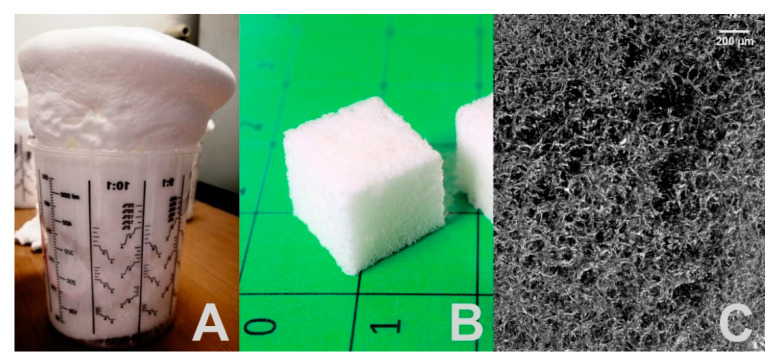
Polyester urethane urea foam: As synthesized (**A**), cube-shaped samples as used for mechanical and thermomechanical characterization (**B**) and µCT image of the foam (**C**).

In the second step, a novel water-blown polyester urethane urea foam was synthesized (Figure 2) following the prepolymer method described in Section 2.1. The one-pot two step reaction resulted in the formation of a porous material characterized by a density of 133 kg·m^−³^. The material in its raw form as well as the samples prepared for the subsequent testing are depicted in Figure 2A,B. For comparison, the typical density range of rigid polyurethane foams used as insulation materials in the construction sector is between 30 and 100 kg·m^−³^ [68,69]. However, the synthesized foam showed typical structural features such as a porosity of 88% like other water-blown polyurethane foams [41,70,71], even at a low blowing agent concentration of 1.3 pphp. This finding was mainly attributed to the manufacturing process. In contrast to the normally used one-shot procedure, the prepolymer method allows a complete transformation of polyols to prepolymers as characterized by NCO-end capped functionalities. This ensured that the otherwise relatively slow reaction between the long-chain polyols and MDI no longer competes with the conversion of the chain extender, cross-linker and water as blowing agent. As a result, a rapid reaction with a cream time of only 3 s to 5 s could be witnessed. The high structural integrity of the polymer at an early stage of the reaction resulted in an improved capability to effectively capture the generated gas and form stable bubbles. 

**Figure 3:**
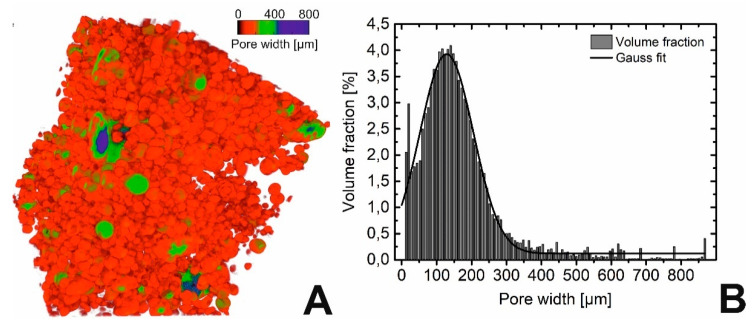
µCT image of PEUU foam after soaking in 1–octanol (**A**), closed cells are colored depending on their diameter) and its evaluation to illustrate the corresponding cell size distribution (**B**).

A detailed insight in the three-dimensional structure of the foam was gained through micro-computed tomography (µCT) measurements (Figure 2C). In order to overcome the limitations of the measurement accuracy and facilitate a reliable statement on the cell size distribution and the interconnectivity of the cells, a second measurement was performed (Figure 3). Hereby the sample was placed for 24 h in 1–octanol to flood the open cells. The resulting image showing the closed cells of the foam is exhibited in Figure 3A. Analysis of the data unveiled a relatively broad pore size distribution for the closed cell with a maximum at around 130 µm (Figure 3B). It can also be deduced from Figure 3B that the majority of the detected pores measured 60 µm to 260 µm in diameter. In addition a larger number of small pores having a width of *d* < 20 µm were present, indicating the high effectiveness of the applied foam stabilizer as nucleating agent, stabilizing gas bubbles in course of foaming. The occurrence of big sized pores is attributed to the undesired coalescence of small bubbles due to their insufficient stability. Another important characteristic of foams is their pore structure. A closed cell structure, as observed in standard insulation materials like rigid PU foams, can be used to capture gases with poor thermal conductivity and thus enhance the insulating effect. A pronounced interconnectivity of the pores, on the other hand, allows limited convection by allowing a certain amount of air exchange through a foam, depending on the pore diameter. The open cell content of the PEUU foam was quantified with 65% from the measurement shown in Figure 3A. To verify this result the buoyancy of the foam was determined employing low viscosity silicon oil. Here, a value of 88% was detected, which might be traced back to the lower viscosity of the fluid and a longer immersion time of 72 h. Thus, two different methods independently confirmed that the PEUU foam was mostly open-celled.

Besides those structural features, the network structure of the polymer also significantly influences the physical behavior of a foam. To estimate the degree of network formation, the gel fraction was determined using a Soxhlet extractor and THF as solvent. The detected gel fraction of 92 ± 1% evidenced the formation of a distinct chemical network structure as introduced through the trifunctional cross-linker DEA. In accordance with the structural features already discussed, the relatively low amount of chemical cross-links was also most comparable with common water-blown flexible PU foams and not with rigid polyurethane foams, which are characterized by much higher values.

### 3.3. Physical Properties

**Figure 4:**
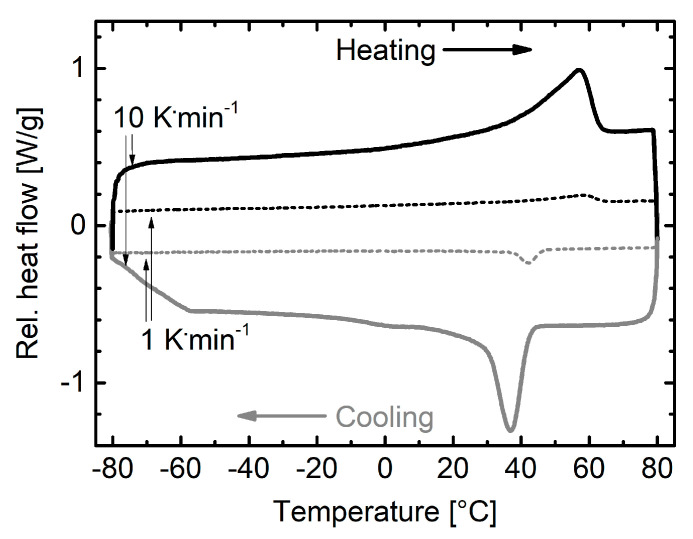
DSC thermogram of PEUU foam during second and third heating and cooling: Influence of the cooling and heating rate (10 K·min^−1^ and 1 K·min^−1^) on the phase transitions of PDA and PBA.

The thermal properties of the foam were investigated by DSC measurements. The corresponding thermogram is depicted in Figure 4. The calorimetric properties of the PEUU were characterized by a melting transition ranging from 20 °C to 65 °C with a peak at 56.5 °C and the corresponding crystallization transition spreading from 45.0 °C to 0.0 °C with a peak at 37.0 °C. The phase transitions were associated with the melting and crystallization of PDA and PBA. Reducing the cooling and heating rate from 10 K·min^−^^1^ to 1 K·min^−^^1^ caused a shift of the crystallization transition to higher temperatures. This time, the onset and offset temperatures of 48.0 °C and 20.0 °C were detected with a peak at 42.5 °C, thus ensuring the complete crystallization above room temperature. In parallel, the melting transition remained nearly unaffected with a high melting enthalpy Δ*H_m_* of 49.8 J·g^−1^. The fact that no separate glass transitions could be detected both for PDA and PBA speaks for the good miscibility of the polyol components, which obviously led to the formation of mixed soft segment phases [72].

As applications in the building industry were being considered for the PEUU foam, the next step was to investigate the insulation capacity. The thermal conductivity *λ* was determined in the permanent shape of the foam to be 0.039 W·(m·K)^−1^. This value is characteristic for insulation materials, whose values are usually in between 0.020 W·(m·K)^−1^ and 0.050 W·(m·K)^−1^ [68,69]. In order to determine the achievable effect with regard to the influence of a volume change of the foam on the thermal resistance, additional tests were carried out on a compressed sample. In this regard, the thermal conductivity was found to increase when reducing the height of the foam from 25.5 mm to 13.4 mm. In parallel, λ rose to 0.052 W·(m·K)^−1^, which corresponds to an increase of about 33%. In turn, the thermal resistance was reduced from 0.66 m²·K·W^−1^ to 0.26 m²·K·W^−1^ and thus by a factor of about 2.5 according Equation (3). In general, different heat flow mechanisms are known to contribute to the thermal conductivity of an insulating foam. Besides the conduction in the solid and in the gas phase, contributions are provided by radiation between cells as well as by convection in the cells [73]. It has been found by other researchers that a higher density of foams leads to decreased values for *λ*, which can be attributed to a reduced level of radiation [74]. By contrast, the opposite effect was verified for the PEUU foam. Obviously, the insulating effect of the cell gases was reduced due to the compression of the foam while at the same time the thermal conductivity through the polymer matrix was more dominant.

In addition, however, the mechanical properties are also significantly affected by structural design. A high proportion of open cells generally reduces the compressive strength but at the same time increases the flexibility and compressibility, which is an important aspect in the view of thermomechanical treatment prior actuation. Anyway, a strong tendency of the polyester phases to crystallize and the covalent cross-linking as initiated by the addition of trifunctional DEA contributed to an enhancement of foam hardness [41]. In detail, the maximum compressive strain as achieved by loading the foam with a compressive stress of 133.6 kPa resulted in a compression of almost 30%. Furthermore, with 63.1 kPa a compressive hardness σ_10%_, defined by the compressive stress which is necessary to achieve a deformation of 10%, was detected.

### 3.4. Thermomechanical Properties

**Figure 5:**
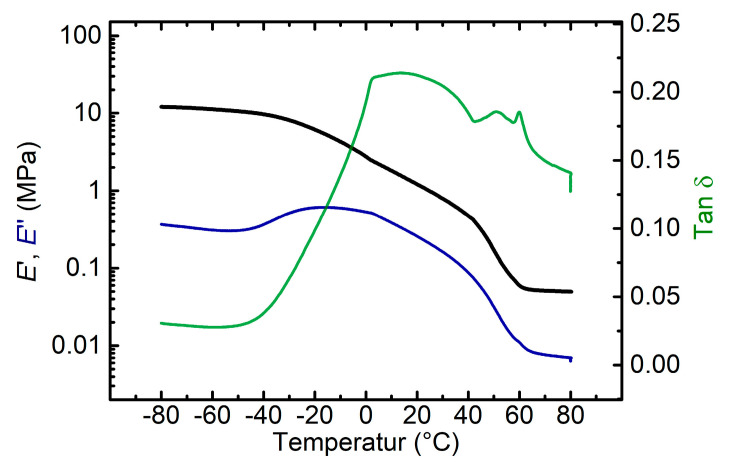
Evolution of storage modulus *E*′ (black color), loss modulus *E*″ (blue color) and tan *δ* (green color) as a function of temperature for the PEUU foam.

DMA measurements were performed to study the thermomechanical behavior of the foam. The storage modulus exhibited a two-step decrease when passing the glass and the melting transition of the PEUU (see Figure 5).

In particular the phase transitions of the polyester-based segments, which later served as actuation segments, were investigated. A broad maximum in the evolution of loss modulus *E*″, which often is used to determine the glass transition temperature *T_g_* of polymers, could be witnessed at −16 °C. The calculation of tan *δ* resulted in a broad signal with no clear peak observable. Such more or less unusual trajectories have been observed in other studies dealing with PU foam as well [75]. The simultaneous presence of a chain extender and a cross-linker as well as of two different polyols differing in chain length led to the formation of a polymeric network with a broad length distribution of the chain segments between the netpoints. This together with a relatively wide distribution of the PEUU molecular weight and the foam structure being composed of both open and closed cells is assumed to result in the broadening of the glass transition temperature. According to the evolution of *E*′, another transition could be observed in between 40 °C and 60 °C, indicating the melting of hitherto crystalline segments of PDA and PBA. As a result, a plateau formed at temperatures above 60 °C and the polymer was transferred from a viscoelastic into a rubber-elastic state. The new state was mainly determined by the hard segment content (HSC) and the degree of cross-linking in the polymer matrix. However, it should not be forgotten that for cellular polymeric materials the response on dynamic mechanical load, as investigated by DMA experiments, is comprised of polymeric and structural effects. Not only molecular composition and cross-linking determine the mechanical properties but also density, cell size and cell structure make a significant contribution [76,77].

**Figure 6:**
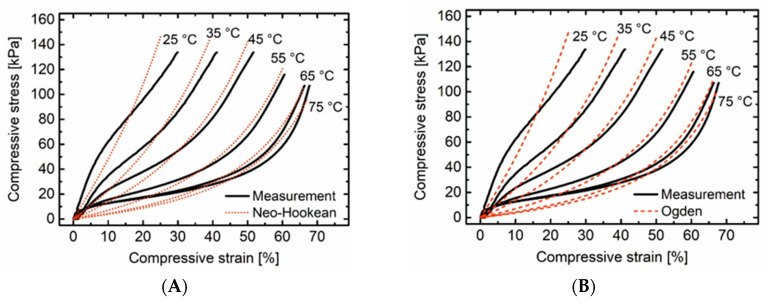
Results of compression tests on PEUU foam at different temperatures and prediction for the evolution of the stress-strain relations as obtained by the Neo-Hookean model (**A**) and the Ogden model (**B**).

In addition, the response of the material to external load at different deformation temperatures *T*_d_ was studied in compression tests (Figure 6).

In any case, the stress-strain relations suggest the existence of three characteristic regions: A narrow linear-elastic region at low levels of strain was followed by a more or less pronounced stress-plateau region and ended with a densification region at higher strains. Exactly this order is characteristic for flexible and semi-rigid polyurethane foams [78]. With increasing *T_d_*, the soft segments were progressively transferred to the molten state, resulting in a flattened curve and a more pronounced plateau area. Based on this data, an effort was made to find a suitable material model that would allow the mechanical response of the PEUU foam to be reliably predicted even under large deformations. Such a model is the first step to digitize the characteristic properties of induvial materials and to incorporate them into the simulation processes of complex systems. For this purpose two widely used hyperelastic models, the Neo-Hookean and the Ogden model, were utilized and the corresponding parameters were determined.

In the Neo-Hookean model, the strain energy function was derived based on molecular chain statistics. As shown in Table 2, this model predicts the stress-strain behavior using *C*_10_ which is a parameter denoting the molecular chain stretching. The Ogden strain energy function was derived based on principal stretches. There are two model parameters for the 1st order Ogden model, which are derived at different temperatures and shown in Table 2. The values of R² are very similar, however, Ogden showed a slightly better correlation with the measurement data.

This is confirmed by Figure 6, where the models showed a very similar prediction of the material response. It is obvious that both models could not accurately track the material behaviour at lower temperatures. As the temperature increased the prediction accuracy was improved. The reason therefore is that the PEUU foam assumed a rubbery state. As a consequence, it experienced higher deformation at similar stresses compared with lower temperatures and exhibited stress-strain relations, which were unproportional and highly non-linear. This allowed the predictions of hyperelastic models to be more accurate. Furthermore, the predictions at the beginning of the loading at lower strains were sometimes inaccurate. The main reason is the order of the models. Employing the models with higher orders might have solved this problem initially, but would have caused extreme instabilities at larger strains which should be avoided. In summary, these models could be used to simulate the behaviour of the PEUU foam especially at higher temperatures and larger deformations.

**Figure 7:**
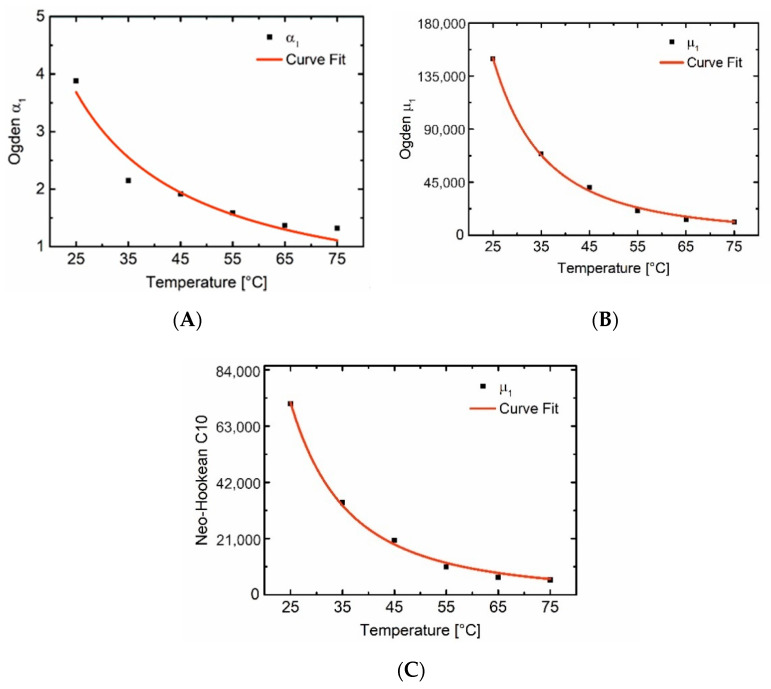
Temperature dependency of model parameters and the fitted power function for Ogden parameters *α*_1_ (**A**) and *µ*_1_ (**B**), and Neo-Hookean parameter *C*_10_ (**C**).

In the following, an approach is presented for the universal prediction of mechanical responses based on temperature changes for this PEUU foam. As stated earlier the model parameters are temperature-dependent. By knowing this correlation, the model parameters can be predicted at various temperatures with which the stress-strain diagrams can be computed. Therefore, the variation of model parameters against the temperature is shown in Figure 7. The power function in the form of

(11) $$y=a\cdot x^{b}$$

could predict this correlation with acceptable accuracy. In Equation (11), *y* is the model parameter, *a* and *b* are material constants shown in Table 3, and *x* is the temperature.

**Figure 8:**
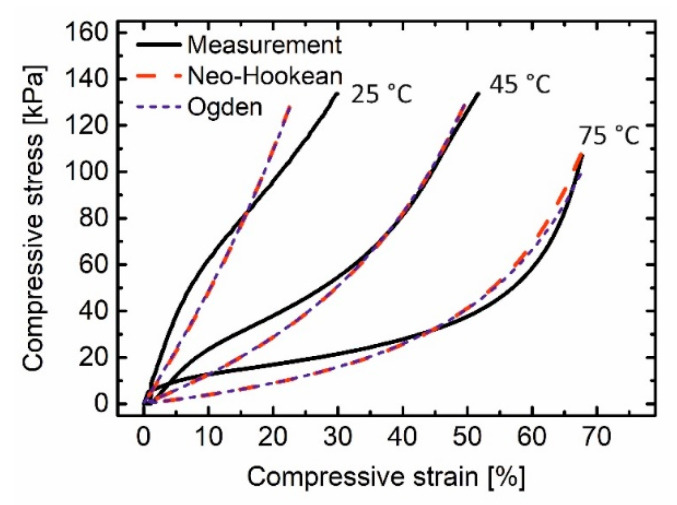
Comparison between the measured stress-strain curves with the ones computed by Neo-Hookean and Ogden model, in which the model parameters were predicted from temperature using the power function.

To verify the accuracy of the correlation equation, the stress-strain diagram was again computed and compared with the measurements at selected temperatures, in which the polymer was in a semicrystalline state (25 °C), an interim state with a lower degree of crystallinity (45 °C), and in a rubbery state (75 °C). In Figure 8, the computed accuracy at 25 °C is not high, since the material response is more elastic than hyperelastic. However, the predictions at higher temperatures are more accurate. Therefore, this universal approach can be used to predict the material response from the temperature. It works for the rubbery state and interim states with higher accuracy compared to the semicrystalline state.

Subsequently, we focused on the two-way shape memory behavior of the foam as strongly affected by thermomechanical treatment. Therefore, a compression-based programming process was selected, enabling a heating-induced expansion opposite to the deformation direction and a cooling-induced contraction in the direction of deformation. The major driving forces of the switching behavior were soft segmental crystallization and melting, both of them were supported by the thermal expansion of the material, which was about 1.9% in between 20 °C and 65 °C as quantified on a sample of pristine PEUU foam in a separate DMA measurement. This value corresponds to a mean thermal expansion coefficient α of 4.3 × 10^−4^ K^−1^. The relatively high value is above the average values of standard PU elastomers of 1.0 × 10^−4^ K^−1^ to 2.5 × 10^−4^ K^−1^ [79,80] and rigid PU foams of approximately 0.5 × 10^−4^ K^−1^ [68]. It is attributed to the melt transition of the polyester segments that occurred in course of heating [27]. In between −20 °C and 20 °C, PDA and PBA were in a semicrystalline state (Figure 4) and the value for *α* was reduced to 1.9 × 10^−4^ K^−1^.

**Figure 9:**
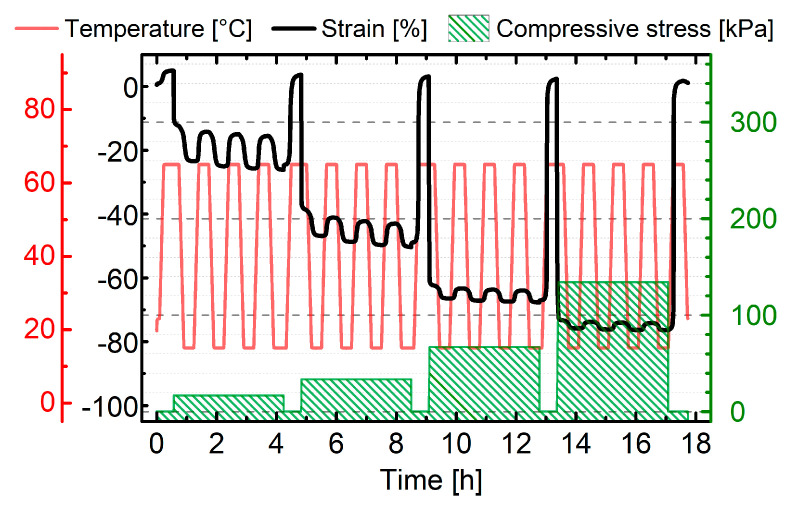
Thermomechanical protocol investigating the influence of compressive stress upon the actuation of the PEUU foam.

The parameter *ε_rev_* was determined for different scenarios to verify the optimal conditions for actuation. First experiments were performed under constant load conditions by varying compressive stress and temperature. Here, highest values for *ε_rev_* were detected at moderate loads. In detail, a thermoreversible actuation of 13.7% was reached in the temperature range of 20 °C to 65 °C when applying a load of 16.8 kPa. Increasing the compressive stress led to actuations of 13.3% for 33.5 kPa, 10.8% for 67.0 kPa and 9.3% for 134.1 kPa together with a continuously growing level for compressive strain (Figure 9). Stresses exceeding 33.5 kPa induced compressive strains higher than 40%, which means that the material was in the densification region. Obviously, foam cells were strongly deformed, thus individual struts and cell walls were getting in touch with each other to a growing extent, enabling a counterintuitive sticking.

Another crucial parameter for actuation is the selected temperature range, since the two-way shape memory effect is mainly driven by the melting and crystallization transitions of the soft segments. In Table 4 the results for the variation of the upper and lower temperatures *T*_high_ and *T*_low_ are summarized. It can be clearly seen that the actuation level under constant load conditions is continuously high with values above 10%. Only when *T*_low_ was raised to 35 °C, *ε_rev_* dropped significantly to 5.7 %, which was obviously due to the incomplete crystallization of the soft segments (see Table 4, entry 3). As expected, a wider temperature range resulted in more distinct actuation [29,81].

In a second step, the transferability of the high actuation capability of the foam to application-related conditions was investigated. In the field of building insulation, new materials need to be broadly applicable, e.g., in different climate zones. In Middle Europe indoor spaces are normally kept at temperatures of 18 °C to 23 °C [82], while, depending on color, exterior façades can reach temperatures of up to 70 °C under intense solar radiation [83]. Therefore, the actuation of the foam in different temperature windows was investigated to evaluate the actuation potentials under conditions of practice (Table 5).

As can be seen in entries 1 and 2 of Table 5, an upper temperature of 45 °C was not sufficient to witness pronounced actuation. This observation was accounted to the incomplete melting and crystallization of the soft segments. In turn, progressive melting resulted in higher actuation values with a maximum occurring for *ε_rev_* at *T*_high_ = 55 °C. In addition, a larger temperature window resulted in stronger actuation, but the differences for *ε_rev_* at a T_high_ of 55 °C were relatively small being less than 1%, especially when *T*_low_ increased from 25 °C to 30 °C. The negative effect of higher values for *T*_low_ exceeding 30 °C could be significantly reduced by increasing the isothermal equilibration time (Table 5, entry 8), which had a similar effect as low heating rates and enabled the actuation segments to sufficiently crystallize. Thermoreversible actuations of 11.4% and 10.8% were found for the temperature combinations *T*_high_ = 55 °C and *T*_low_ = 25 °C same as for *T*_high_ = 55 °C and *T*_low_ = 30 °C, thus representing a very good starting point for the development of novel thermoresponsive insulation materials.

**Figure 10:**
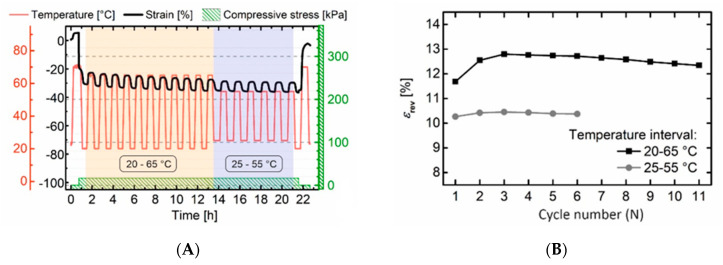
Thermomechanical protocol investigating the influence of *T*_high_ and *T*_low_ upon actuation of PEUU foam under constant load conditions in a multiple cycle experiment: Stress-strain-temperature protocol (**A**) and evolution of the associated actuation (**B**).

Since the stability of actuation is also an important parameter from a user’s perspective, a multiple cycling experiment was carried out (see Figure 10). In this connection, actuation turned out to be only slightly decreasing from the second to the eleventh cycle as evidenced by values for *ε_rev_* of 12.6 ± 0.2% in the temperature range from 20 °C to 65 °C (see Figure 10B). Reducing the temperature window for actuation to the area Δ*T* = 25 °C to 55 °C led to a slightly lower actuation of 10.4 ± 0.1% in five more thermal cycles (see Figure 10B). Considering the fact that the experiments for the second temperature window were directly carried out after running eleven thermal cycles, it can be stated that in a total an actuation was witnessed in 17 cycles. In course of multiple cycling an increase in compressive strain level as associated with creeping was observed. However, its declining slope indicates that actuation became more and more stable. Additional experiments with an extension of the number of cycles would be necessary to finally prove the stability of the effect.

As a further step, bidirectional actuation under load-free conditions was examined. Thermal cycling with a stepwise increase of *T*_high_ was performed to identify those conditions, under which a maximum actuation occurred. The results are provided in Table 6. It was found that the upper temperatures close to the melting peak temperature of 56.5 °C triggered the recovery of a large part of strain, thus reducing the potential of actuation (Table 6). For all loading stresses *σ_load_* applied, the foam performed quite well at temperatures *T*_high_ of 42 °C, 45 °C and 48 °C. In particular, a maximum actuation with a value of 10.4% could be detected at a *T*_high_ of 45 °C after loading with a stress of 33.3 kPa (Table 6, entry 7). A similarly good result of 9.7% was achieved when selecting a *T*_high_ of 48 °C and a σ_load_ of 50.0 kPa (Table 6, entry 13).

**Figure 11:**
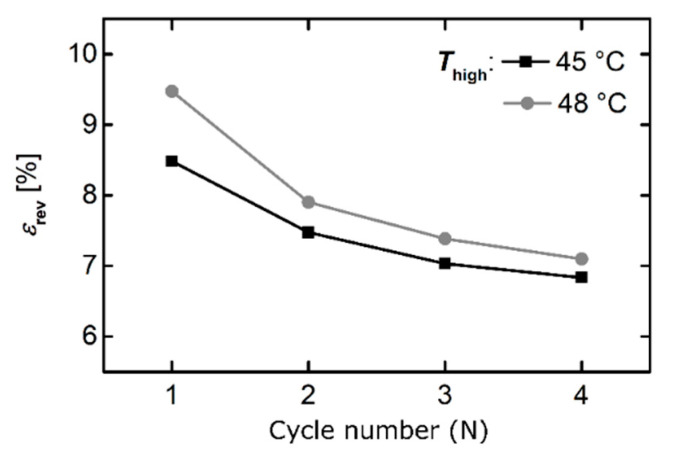
Durability of actuation for the PEUU foam under load-free conditions depending on *T*_high_.

The durability of actuation was verified in multiple cycling experiments as illustrated in Figure 11. Independent of the choice of *T*_high_**,** a drop in actuation from the first to the second cycle was observed followed by a slow but continuous decrease in the value for *ε_rev_*. Anyway, its decrease in value was lower from cycle to cycle. 

### 3.5. Demonstrator

**Figure 12:**
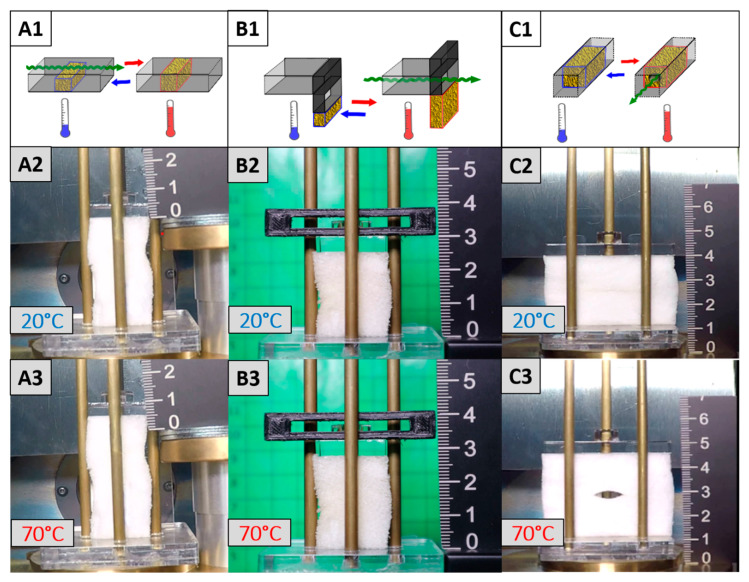
Schematic representation for the foam actuator concept of first (**A1**), second (**B1**) and third generation (**C1**) and pictures of the corresponding demonstrators in their boundary states at *T*_low_ (**A2**,**B2**,**C2**) and *T*_high_ (**A3**,**B3**,**C3**). For the sake of simplicity, the weight on the foam is not shown.

Having evidenced the excellent two-way shape memory properties of the water-blown foam in particular under constant load conditions, we transferred the results to application-related demonstrators. With the aim to develop a programmable material, which is able to be repeatedly and solely controlled by the environmental conditions to enable a switching of convection from “ON” to “OFF” and vice versa, three generations of demonstrators were developed (Figure 12). In the first generation concept (Figure 12A1) an increase in temperature triggered the closing of an aperture whereas a temperature decrease resulted in an opening of the same. Such a design could be employed to discontinue a heated gas stream in order to avoid overheating. The second generation demonstrator (Figure 12B1) was constructed to open a slot outside of the foam on heating and close it on cooling, which can be utilized in thermal regulation applications. The third generation concept (Figure 12C1) had a switchable opening inside the foam, solely enabling convection at elevated temperature. This design led to a certain simplification of the construction and can also be used as a component in future insulation systems. Especially the last two designs can be useful concepts in adaptive insulation systems. They can be easily implemented without restricting the design of the building envelope’s outer surface and are able to provide a self-regulated overheating protection by having two different modes–insulating and dissipative. In the first mode, a layer of dead air provides good insulation at low temperatures, while an increased temperature inside the wall associated with a high heat input from intensive solar radiation initiates an opening of the foam to allow air to flow. This transforms the stagnant air into a moving layer, enabling heat transfer by convection and has thus a dissipative effect, similar to a ventilated façade. To address this, the PEUU foam was exposed to a constant external load of 19.6 kPa, before the temperature was cycled in between 20 °C and 70 °C. As a result, bidirectional actuation with thermoreversible strains of 12.5% for the first and third generation demonstrators (Figure 12A2,A3,C2,C3) and of 12% for the second generation design (Figure 12B2,B3) could be verified.

### 3.6. Summary

A novel polyester urethane urea (PEUU) foam was successfully synthesized and its actuation behavior investigated. It could be demonstrated that a thermomechanical treatment enables the switching of the foam both under constant load and in a load-free state. Furthermore, a strong reversible actuation in a significantly narrower temperature interval compared to previous studies could be achieved (Table 7). Beyond two-way shape memory properties, the presented foam showed good insulation behavior. The simulation of the mechanical properties of the foam using the hyperplastic model Ogden led to a good agreement with the experimental data as obtained from mechanical testing. Together with the successful proof-of-concept for programmable apertures, an important step toward a system demonstrator, which can later be subjected to field tests, was made.

## 4. Conclusions

In the future, a paradigm shift can be initiated by understanding the programming of a material as the programming of a functionality. The internal structure of the PEUU foam is such that the material properties and behavior change reversibly according to a program. This is achieved by permanently programming the material’s response to a temperature signal into the material structure. In addition to the internal structure of foams, the design layout can also contribute significantly to the development of new applications. This makes it possible to produce completely new components with programmable heat transport properties that can be used in a wide variety of contexts. First approaches for the building industry were presented and discussed. The most significant opportunity could be the use as innovative insulating material in adaptive building envelopes, in which the foam makes a contribution to improve energy efficiency and living comfort. It could also be attractive from an economic point of view when reducing the costs for the heating and cooling of buildings and make a contribution to the energy turnaround. Apart from that, industries such as automotive or soft robotics can also be addressed. If one takes into account that neither control electronics, nor cables or other technical equipment are required in such concepts, the foam with its self-sufficient material behavior appears all the more advantageous. 

## Figures and Tables

**Table 1:** Results of functional group analysis after titration and thermal properties of polyester diols as determined from DSC measurements.

Polyester	Hydroxy Value (mg(KOH)·g^−^^1^)	Acid Value(mg(KOH)·g^−^^1^)	*M_n_*^1^ (g·mol^−^^1^)	*T_m,peak_*^2^ (°C)	Δ*H_m_* ^3^ (J·g^−^^1^)	*T_c,peak_*^4^ (°C)	Δ*H_c_* ^5^ (J·g^−^^1^)
PDA	32.0	0.2	3500	69	139	57	142
PBA	50.5	0.1	2220	47/52	87	32	75

^1^ Number average molecular weight, ^2^ peak temperature of melting, ^3^ melting enthalpy, ^4^ peak temperature of crystallization, ^5^ crystallization enthalpy.

**Table 2:** The model parameters (see Section 2.5) of 1st order Ogden and Neo-Hookean strain energy function.

Temperature (°C)	Ogden		R²	Neo-Hookean	R²
	*µ*_1_	*α*_1_		*C*_10_	
25	149,753.0	3.9	0.9575	71,360.0	0.9589
35	69,164.4	2.1	0.9890	34,424.3	0.9889
45	40,593.3	1.9	0.9966	20,340.7	0.9966
55	20,800.7	1.6	0.9984	10,455.4	0.9976
65	13,008.8	1.4	0.9961	6480.9	0.9933
75	11,239.6	1.3	0.9929	5557.2	0.9897

**Table 3:** Material constants *a* and *b* for the power equation denoting the correlation between temperature and Ogden and Neo-Hookean parameters. The correlation coefficient (R^2^) is between computed model parameters and the power equation.

Model Parameter	*a*	*b*	R²
Ogden *µ*_1_	2.96 × 10^8^	−2.36	0.9982
Ogden *α*_1_	124 × 06	−1.09	0.9456
Neo-Hookean *C*_10_	1.10 × 10^8^	−2.28	0.9973

**Table 4:** Bidirectional actuation of PEUU foam under constant load conditions (σ = 16.8 kPa) for different temperature ranges.

Entry	*T*_low_ (°C)	*T*_high_ (°C)	*t_iso_*^1^ (min)	Δ*T* ^2^ (°C)	*ε_T_*_low_^3^ (%)	*ε_rev_* (%)
1	15	65	20	50	25.6	12.2
2	25	65	20	40	26.2	11.5
3	35	65	20	30	22.5	5.7
4	20	55	20	35	28.0	10.6
5	20	65	20	45	28.2	12.2
6	20	75	20	55	28.8	12.8

^1^ Isothermal equilibration time at *T*_low_, ^2^ difference between *T*_high_ and *T*_low_, ^3^ compressive strain at *T*_low_.

**Table 5:** Bidirectional actuation of PEUU foam under constant load conditions (*σ* = 16.8 kPa) in application-adapted temperature ranges.

Entry	*T*_low_ (°C)	*T*_high_ (°C)	*t_iso_*^1^ (min)	Δ*T* ^2^ (°C)	*ε_T_*_low_^3^ (%)	*ε_rev_* (%)
1	20	45	30	25	38.7	3.9
2	25	45	30	20	38.7	3.3
3	20	50	30	30	38.5	9.3
4	25	50	30	25	38.6	8.7
5	30	50	30	20	38.3	7.3
6	25	55	40	30	34.0	11.4
7	30	55	50	25	34.5	10.8
8	35	55	60	20	33.8	9.0

^1^ Isothermal equilibration time at *T*_low_, ^2^ difference between *T*_high_ and *T*_low_, ^3^ compressive strain at *T*_low_.

**Table 6:** Influence of loading stress *σ_load_* during thermomechanical treatment on bidirectional actuation of the PEUU foam under load-free conditions (*T*_low_ = 20 °C).

Entry	*σ_load_* (kPa) ^1^	*T*_high_ (°C)	ε*_T_*_low_^2^ (%)	ε*_T_*_high_^2^ (%)	*ε_rev_* (%)
1	16.7	42	32.4	28.3	6.1
2	16.7	45	21.2	15.4	7.4
3	16.7	48	12.7	7.9	5.5
4	16.7	51	8.7	4.8	4.2
5	16.7	54	6.7	3.2	3.7
6	33.3	42	55.3	51.7	8.2
7	33.3	45	37.6	31.0	10.6
8	33.3	48	16.9	11.4	6.7
9	33.3	51	9.8	5.5	4.7
10	33.3	54	7.3	3.6	4.0
11	50.0	42	65.6	64.2	4.1
12	50.0	45	61.8	58.7	7.9
13	50.0	48	29.3	22.5	9.7

^1^ Loading stress in course of compression during programming, ^2^ compressive strain values referred to the length of sample at *T_d_* without load.

**Table 7:** Comparison of the PEUU foam with other ones from the literature with regard to their production methods and actuation properties.

Polymer Type	Foaming Process	*ε_rev_* (%)	*T*_low_ (°C)	*T*_high_ (°C)	Actuation Mode	Reference
PCL-co-PEG	porogen leaching	15	−20	80	compression/constant load	[39]
Crosslinked PCL	incorporation of glass microspheres	N/A	N/A	N/A	compression/load-free	[40]
Crosslinked PCL	incorporation of glass microspheres	>10	0	59	elongation */load-free	[40]
PCL-PU	water-blown	12	10	50	compression/load-free	[41]
PEUU	water-blown	>13	20	65	compression/constant load	current work
PEUU	water-blown	>10	25	55	compression/load-free	current work

* Samples were elongated in course of programming to strains above 100%.
